# The missing discipline in AI: a call for behavioural science

**DOI:** 10.12688/wellcomeopenres.25922.1

**Published:** 2026-02-26

**Authors:** Paul M Sacher, Susan Michie, Oliver P Hauser, Antoine Ferrère, Samuel Salzer, Amy Rodger, Jana Schaich Borg, Ganna Pogrebna, Susan A Murphy

**Affiliations:** 1 Imperial College London Faculty of Medicine, London, England, UK; 2 Behavioral AI Institute, Paris, N/A, 70123, France; 3 University College London Faculty of Brain Sciences, London, England, UK; 4 University of Exeter Department of Economics, Exeter, England, UK; 5 The University of Edinburgh School of Social and Political Science, Edinburgh, Scotland, UK; 6 Duke University Social Science Research Institute, Durham, North Carolina, USA; 7 Queen's University Belfast, Belfast, Northern Ireland, UK; 8 The Alan Turing Institute, London, UK; 9 The University of Sydney, Sydney, New South Wales, Australia; 10 Harvard University Department of Statistics, Cambridge, Massachusetts, USA; 11 Kempner Institute for the Study of Natural and Artificial Intelligence, Boston, USA

**Keywords:** Behavioural science, AI governance, human–AI interaction, behavioural safety, responsible AI, digital harms

## Abstract

Artificial intelligence systems increasingly shape how people think, feel, and act, particularly in high-impact domains such as healthcare, education, and social support. While significant attention has been paid to technical performance, bias, privacy, and security, the behavioural impacts of AI systems remain under-evaluated and under-governed.

This Open Letter outlines why behavioural science should be treated as a core component of responsible AI practice. Even when technically accurate, AI systems can influence behaviour in ways that are harmful, misleading, or misaligned with people’s interests. These effects are often predictable consequences of repeated human–AI interaction, yet they are rarely assessed systematically.

We describe where behavioural risks arise in practice and outline what good practice looks like, drawing on established behavioural science methods. We then propose practical steps for funders, researchers, and developers to embed behavioural expertise and behavioural evaluation across the AI lifecycle.

Addressing behavioural impacts is essential to ensuring that AI systems are not only effective, but safe, trustworthy, and appropriate for real-world use.

## Plain Language summary

Artificial intelligence systems are increasingly used in areas that affect people’s lives directly, such as healthcare, education, and emotional support. These systems do more than provide information. They can influence how people think, make decisions, feel about themselves, and behave over time.

Most efforts to make AI safe focus on technical accuracy, bias, or security. Much less attention is paid to behavioural effects, such as whether an AI system encourages unhealthy dependence, undermines confidence, increases anxiety, or subtly pushes people toward certain choices.

Behavioural science studies how people think, feel, and act. It offers well-established methods for understanding influence, motivation, trust, and decision making. Despite this, behavioural expertise is often missing from AI development and evaluation, especially outside academic settings.

This Open Letter argues that behavioural science should be treated as a core part of responsible AI, not an optional extra. We outline where behavioural risks can arise, why they matter, and how they can be addressed using existing tools and expertise.

## Introduction

Artificial intelligence systems increasingly interact directly with people, including through conversational agents, recommender systems, and decision-support tools used in healthcare, education, finance, and social support. In these contexts, AI systems do more than complete tasks or provide information. They influence how people interpret information, make decisions, regulate emotions, and form habits over time. ^1, 2^

Much of the current work on responsible AI focuses on technical performance, bias, privacy, and security. These areas are important. However, they do not fully address how AI systems affect behaviour in real-world use. Systems may perform well on technical metrics while still shaping behaviour in ways that undermine people’s interests or wellbeing. ^3^

Behavioural science provides established methods for understanding influence, motivation, trust, and decision making. Despite this, behavioural expertise is often underrepresented in the development, evaluation, and governance of AI systems, particularly outside academic research settings. Behavioural risks are therefore often identified late, addressed inconsistently, or not assessed at all. ^3^

By behavioural science, we refer to disciplines that systematically study how people think, feel, decide, and act, including psychology, behavioural economics, and decision sciences. In this context, behavioural science focuses on how AI systems influence human cognition, emotion, motivation, and behaviour through repeated interaction. This is distinct from research on agent behaviour within AI systems themselves, which examines how models learn, adapt, or optimise over time.

This Open Letter argues that behavioural science must play a central role in responsible AI practice. This includes systematic evaluation of behavioural impacts and integration of behavioural expertise across the AI lifecycle, especially in high-impact domains where AI systems are used repeatedly and at scale.

## What is currently missing

Although AI systems are known to influence human behaviour, behavioural considerations are rarely treated as a core requirement in AI development and deployment. Behavioural expertise is often absent from project teams or involved only informally and late in the process. ^4^

Evaluation practices typically prioritise task success, accuracy, efficiency, or user satisfaction. These measures are useful, but they do not capture how AI systems shape behaviour over time. They rarely assess effects such as over-reliance, reduced confidence, increased anxiety, distorted decision making, or unintended changes in motivation. ^3^ These effects often arise through well-documented behavioural mechanisms, including automation bias, inappropriate trust calibration, anthropomorphism, emotional dependency, and uncritical reinforcement of user beliefs in personalised systems.

When behavioural risks are considered, they are often framed as secondary or ethical edge cases. This underestimates the extent to which behavioural effects are predictable outcomes of repeated human–AI interaction, particularly when systems are used frequently or by large numbers of people. ^5^

Behavioural science offers well-established tools to identify, measure, and mitigate these effects. However, these tools are not consistently embedded in AI research pipelines, funding requirements, or governance frameworks. This creates a gap between technical performance and real-world impact. Closing this gap requires treating behavioural science as foundational infrastructure for responsible AI, not as an optional addition.

## Where behavioural risks arise in practice

In this context, behavioural safety refers to the extent to which an AI system avoids causing predictable harms to people’s decision making, motivation, emotional wellbeing, sense of agency, or help-seeking behaviour over time.

Behavioural risks do not arise only from incorrect or biased outputs. They can emerge even when AI systems provide accurate information and operate as intended. These risks are shaped by how systems communicate, personalise responses, and position themselves within decision making processes. ^6^

People frequently interact with AI systems as if they were social actors, attributing intent, authority, or understanding even while recognising their artificial nature. Such interactions are shaped by social heuristics and cognitive biases, including anthropomorphism, default effects, and social mimicry, which can amplify influence in subtle but consequential ways.

Language and framing play an important role. The tone, confidence, and style of an AI system influence how people interpret advice and how much authority they assign to it. Small differences in wording can affect motivation, emotional response, and willingness to act. ^6^

Personalisation introduces further risks. Systems that adapt to people’s behaviour may unintentionally reinforce existing beliefs, habits, or vulnerabilities. Over time, this can narrow perspectives, increase dependence, or reduce people’s sense of agency, particularly in contexts involving health or long-term behaviour change. ^7^

Feedback and reinforcement also matter. AI systems often provide reassurance, encouragement, or correction. While these features can be helpful, they can also influence self-perception, confidence, and emotional regulation. Without careful design and evaluation, systems may normalise unhealthy patterns or discourage appropriate help-seeking. ^3^

These risks are most pronounced in high-impact domains where people interact with AI systems repeatedly and place trust in their guidance. In such settings, behavioural effects may accumulate gradually and remain invisible to standard performance metrics. ^1, 3^

## What good practice looks like

Addressing behavioural risks does not require fundamentally new approaches to AI development or governance. It requires integrating behavioural considerations systematically across the AI lifecycle, using existing expertise and methods. ^8^

A key shift is moving beyond task success toward what might be described as psychological competence: an AI system’s ability to respond in ways that are emotionally appropriate, behaviourally responsible, and calibrated to people’s needs and context over time. This includes how systems shape trust, influence confidence, support agency, and affect decision making across repeated interactions.

Good practice begins at the design stage. Behavioural assumptions about people’s goals and decision making should be made explicit and tested, rather than embedded implicitly in system prompts or interaction design. Early involvement of behavioural expertise helps identify where design choices may influence behaviour in unintended ways. ^4^

During development and training, behavioural considerations should inform how systems are instructed, fine-tuned, and evaluated. This includes attention to language, feedback styles, and personalisation strategies, as well as how systems respond to uncertainty or repeated use. ^9^

Evaluation is essential, but it should be proportionate to risk and intended use. Lightweight behavioural assessments may be appropriate for low-risk systems, while AI systems used in high-impact domains may require more structured and longitudinal evaluation. In all cases, behavioural assessment should be deliberate and theory-informed, rather than ad hoc. ^3^

Good practice also involves ongoing monitoring. Behavioural impacts may change over time as people adapt to a system or as the system evolves. Regular review can help identify emerging risks early and reduce the likelihood of harm. ^3^

Governance structures should recognise behavioural safety as part of responsible AI. Clear accountability, access to appropriate expertise, and transparency about behavioural evaluation support the development of AI systems that are more trustworthy and effective in real-world use. ^9^

## Recommendations and next steps

We call on developers, funders, researchers, and institutions to take the following steps to ensure that behavioural considerations are treated as core infrastructure for AI development and governance.

1. **Treat behavioural impact as a core evaluation requirement (developers, deployers).**
AI systems should be evaluated not only for technical performance, but also for their effects on behaviour, decision making, motivation, trust, and emotional wellbeing, particularly in high-impact or repeated-use contexts.
2. **Embed behavioural expertise across the AI lifecycle (research teams, organisations).**
Behavioural scientists should be involved throughout design, development, evaluation, and governance, rather than consulted only during post-hoc review or ethical oversight.
3. **Fund behavioural evaluation explicitly (funders).**
Research funders should allocate dedicated resources for behavioural assessment and ongoing monitoring, instead of relying on unfunded, ad hoc, or discretionary effort.
4. **Develop shared behavioural benchmarks and metrics (research community).**
The field would benefit from common, theory-informed approaches to assessing behavioural effects, particularly for AI systems designed to interact with people over time.
5. **Support interdisciplinary norms and training (institutions, funders).**
Institutions should actively encourage collaboration between AI researchers, behavioural scientists, clinicians, and domain experts as a routine component of responsible AI practice.

## Conclusion

AI systems that interact with people inevitably influence behaviour. These effects are not incidental, nor limited to cases of technical error or misuse. They are a predictable consequence of how AI systems communicate, personalise responses, and position themselves within human decision making.

Behavioural science offers practical, well-established tools to understand and mitigate these effects. Treating behavioural considerations as core infrastructure, rather than as an optional or downstream concern, supports the development of AI systems that are safer, more trustworthy, and more effective in real-world settings.

Funders, researchers, and developers play a critical role in shaping norms, incentives, and expectations around responsible AI. Embedding behavioural expertise and behavioural evaluation early is likely to be more effective, and less costly, than responding to harm after deployment.

We hope this Open Letter contributes to a broader and more systematic approach to behavioural safety in AI and encourages continued collaboration across disciplines to ensure that AI systems are designed and governed with human behaviour firmly in view.

## Disclaimer

The views expressed in this article are those of the author(s). Publication in Wellcome Open Research does not imply endorsement by Wellcome.

## Data Availability

No data are associated with this article.
