# The missing discipline in AI: a call for behavioural science

**DOI:** 10.12688/wellcomeopenres.25922.2

**Published:** 2026-06-15

**Authors:** Paul M Sacher, Susan Michie, Oliver P Hauser, Antoine Ferrère, Samuel Salzer, Amy Rodger, Jana Schaich Borg, Ganna Pogrebna, Susan A Murphy

**Affiliations:** 1 Imperial College London Faculty of Medicine, London, England, UK; 2 Behavioral AI Institute, Paris, France; 3 University College London Faculty of Brain Sciences, London, England, UK; 4 University of Exeter Department of Economics, Exeter, England, UK; 5 The University of Edinburgh School of Social and Political Science, Edinburgh, Scotland, UK; 6 Duke University Social Science Research Institute, Durham, North Carolina, USA; 7 Queen's University Belfast, Belfast, Northern Ireland, UK; 8 The Alan Turing Institute, London, UK; 9 The University of Sydney, Sydney, New South Wales, Australia; 10 Harvard University Department of Statistics, Cambridge, Massachusetts, USA; 11 Kempner Institute for the Study of Natural and Artificial Intelligence, Boston, USA

**Keywords:** Behavioural science, AI governance, human–AI interaction, behavioural safety, responsible AI, digital harms

## Abstract

Artificial intelligence systems increasingly shape how people think, feel, and act, particularly in high-impact domains such as healthcare, education, and social support. While significant attention has been paid to technical performance, bias, privacy, and security, the behavioural impacts of AI systems remain under-evaluated and under-governed.

This Open Letter outlines why behavioural science should be treated as a core component of responsible AI practice. Even when technically accurate, AI systems can influence behaviour in ways that are harmful, misleading, or misaligned with people’s interests. These effects are often predictable consequences of repeated human–AI interaction, yet they are rarely assessed systematically.

We describe where behavioural risks arise in practice and outline what good practice looks like, drawing on established behavioural science methods. We then propose practical steps for funders, researchers, and developers to embed behavioural science expertise and behavioural evaluation across the AI lifecycle, including behavioural testing pipelines, longitudinal evaluation, and interdisciplinary governance.

Addressing behavioural impacts is essential to ensuring that AI systems are not only effective, but safe, trustworthy, and appropriate for real-world use.

## Plain Language summary

Artificial intelligence systems are increasingly used in areas that affect people’s lives directly, such as healthcare, education, and emotional support. These systems do more than provide information. They can influence how people think, make decisions, feel about themselves, and behave over time.

Most efforts to make AI safe focus on technical accuracy, bias, or security. Much less attention is paid to behavioural effects, such as whether an AI system encourages unhealthy dependence, undermines confidence, increases anxiety, or subtly pushes people toward certain choices.

Behavioural science studies how people think, feel, and act. It offers well-established methods for understanding influence, motivation, trust, and decision making. Despite this, behavioural expertise is often missing from AI development and evaluation, especially outside academic settings.

This Open Letter argues that behavioural science should be treated as a core part of responsible AI, not an optional extra. We outline where behavioural risks can arise, why they matter, and how they can be addressed using existing tools and expertise.

## Introduction

Artificial intelligence systems increasingly interact directly with people, including through conversational agents, recommender systems, and decision-support tools used in healthcare, education, finance, and social support. In these contexts, AI systems do more than complete tasks or provide information. They influence how people interpret information, make decisions, regulate emotions, and form habits over time. ^1^ ^–^ ^3^

These behavioural effects are increasingly recognised in empirical research, including studies showing that AI systems can influence user beliefs and decision-making. ^2^ ^–^ ^4^ In such settings, the relevant unit of evaluation is not only the model in isolation, but the human-AI interaction and its effects on users.

Much current work on responsible AI focuses on technical performance, bias, privacy, and security. These areas are important. However, they do not fully address how AI systems affect behaviour in real-world use. Systems may perform well on technical metrics while still shaping behaviour in ways that undermine people’s interests or wellbeing. ^3^ ^–^ ^6^

From a behavioural science perspective, human-facing AI systems can often function as behavioural interventions deployed at scale, shaping how users interpret information, regulate emotions, and make decisions over time. Behavioural science provides established methods for understanding influence, motivation, trust, and decision-making. Despite this, behavioural science expertise is often underrepresented in the development, evaluation, and governance of AI systems, particularly outside academic research settings. ^4^ ^–^ ^8^ Behavioural risks are therefore often identified late, addressed inconsistently, or not assessed at all.

By behavioural science, we refer to disciplines that systematically study how people think, feel, decide, and act, including psychology, behavioural economics, and decision sciences. In this context, behavioural science focuses on how AI systems influence human cognition, emotion, motivation, and behaviour through repeated interaction. This is distinct from research on agent behaviour within AI systems themselves, which examines how models learn, adapt, or optimise over time.

Some may argue that existing fields such as human-computer interaction, user experience research, and AI ethics already address these concerns. These disciplines provide important methods for evaluating usability, trust, and interaction quality. ^7^ Behavioural science complements these approaches by providing predictive and explanatory frameworks for understanding how repeated interactions shape cognition, emotion, motivation, and behaviour, particularly in systems designed for ongoing interaction.

### What is currently missing

Although AI systems are known to influence human behaviour, behavioural impact assessment is rarely treated as a core requirement in AI development and deployment. Behavioural science expertise is often absent from project teams or involved only informally and late in the process. ^5^ ^–^ ^8^

Evaluation practices typically prioritise task success, accuracy, efficiency, or user satisfaction. These measures are useful, but they do not capture how AI systems shape behaviour over time. They rarely assess effects such as over-reliance, reduced confidence, increased anxiety, distorted decision-making, or unintended changes in motivation.

Behavioural science offers well-established tools to identify, measure, and mitigate these effects, particularly where AI systems function as interventions or influence decision-making over time. These tools span theory, specification, and evaluation.

They include theories of behaviour and behaviour change, such as COM-B and the Behaviour Change Wheel; taxonomies for specifying intervention content, such as the Behaviour Change Technique Taxonomy; frameworks for developing and evaluating complex interventions; experimental methods suited to digital environments, such as micro-randomised trials and ecological momentary assessment; and emerging behavioural data science approaches for evaluating AI systems and human-AI interaction. ^9^ ^–^ ^14^

However, these approaches are not consistently embedded within AI research pipelines, funding structures, or governance frameworks, despite their direct relevance to systems intended to shape or influence human behaviour. As a result, there remains a persistent gap between technical performance metrics and real-world behavioural impact. Behavioural science should therefore be treated as foundational infrastructure for responsible AI, rather than as an optional or downstream addition.

### Where behavioural risks arise in practice

Behavioural safety refers to the extent to which an AI system identifies, reduces, and monitors predictable risks to users’ decision-making, motivation, emotional wellbeing, sense of agency, and help-seeking behaviour over time.

Behavioural risks do not arise only from incorrect or biased outputs. They can emerge even when AI systems provide accurate information and operate as intended. These risks are shaped by how systems communicate, personalise responses, and position themselves within decision-making processes over time.

These risks are not only theoretical; they have been documented in empirical and experimental studies. For example, research on automation bias in human-AI collaboration shows that users may become over-reliant on automated advice, reducing independent judgement. ^15^ In mental health contexts, reviews of large language models and conversational systems highlight unresolved clinical risks, including inconsistent or hallucinatory outputs, potential reinforcement of harmful beliefs, and delayed appropriate professional intervention. ^16^ Similarly, personalised recommender systems can narrow users’ information environments and reinforce existing beliefs, while psychological targeting can use inferred profiles to influence attitudes, emotions, or behaviours at scale. ^17^ ^,^ ^18^

People frequently interact with AI systems as if they were social actors, attributing intent, authority, or understanding even while recognising their artificial nature. Such interactions are shaped by anthropomorphism and related social heuristics, which can amplify influence in subtle but consequential ways. ^8^ ^,^ ^19^

Language and framing play an important role. The tone, confidence, and style of an AI system can shape how people interpret advice and how much authority they assign to it. In conversational and health-related contexts, small differences in content or tone can alter responses and persuasive effects, with implications for motivation, emotional response, and willingness to act. ^3^ ^,^ ^8^ ^,^ ^16^ ^,^ ^19^

Personalisation introduces further risks. Systems that adapt to people’s behaviour may unintentionally reinforce existing beliefs, habits, or vulnerabilities. Over time, this can narrow perspectives and, in social and psychological systems, increase dependence or reduce people’s sense of agency. ^8^ ^,^ ^17^ ^,^ ^18^

Feedback and reinforcement also matter. AI systems may provide reassurance, encouragement, or correction. While these features can be helpful, they can also influence self-perception, confidence, and emotional regulation. Without careful design and evaluation, systems may risk reinforcing harmful beliefs, normalising unhealthy patterns, or delaying appropriate professional intervention. ^16^

These behavioural effects may accumulate gradually through repeated interaction and remain invisible to standard technical performance metrics, particularly in systems used frequently or over long periods.

### What good practice looks like

Addressing behavioural risks requires integrating behavioural science expertise and behavioural evaluation systematically across the AI lifecycle, using existing expertise and methods. The depth and intensity of behavioural evaluation should be proportionate to system risk, deployment intensity, and domain sensitivity.

A key shift is moving beyond task success towards what might be described as psychological competence. This term is used here as a provisional organising concept, not as a validated construct, for how AI-generated interactions shape user cognition, emotional regulation, and behavioural decision-making through interaction mechanisms such as framing, tone, feedback, and perceived authority. Existing evaluation approaches only partially capture these behavioural interaction dynamics and rarely assess them explicitly, leaving important interaction risks under-examined.

Good practice begins at the design stage. Behavioural assumptions about users’ goals and decision-making should be made explicit and tested. Early involvement of behavioural science expertise helps identify where design choices may influence behaviour in unintended ways.

During development and training, behavioural science expertise and behavioural evaluation should inform how systems are instructed, fine-tuned, and evaluated. This includes attention to language, feedback styles, personalisation strategies, and how systems communicate uncertainty.

Evaluation should include structured scenario testing, simulation approaches using synthetic or simulated users as exploratory stress-testing methods, and, where appropriate, longitudinal assessment of behavioural effects across repeated interactions.

Good practice also involves ongoing monitoring. Behavioural impacts may change over time as people adapt to a system, as deployment contexts shift, or as systems evolve.

Governance structures should recognise behavioural safety as part of responsible AI. Clear accountability, access to appropriate expertise, and transparency about behavioural evaluation support the development of AI systems that are more trustworthy and effective in real-world use.

### Recommendations and next steps

1. **Treat behavioural impact as a core evaluation requirement (developers, deployers).**
Evaluate AI systems for behavioural effects, not only technical performance. This includes effects on decision-making, motivation, trust, and emotional wellbeing, particularly in high-impact or repeated-use contexts.
Before deployment, use behavioural testing pipelines with structured user scenarios to identify behavioural failure modes. Where appropriate, simulation approaches, such as synthetic or simulated users, may be used as exploratory stress-testing methods alongside human-centred evaluation where systems meaningfully affect users.
For systems intended for repeated interaction, longitudinal evaluation is needed to assess how behavioural effects accumulate over time.
2. **Embed behavioural science expertise across the AI lifecycle (research teams, organisations).**
Involve behavioural scientists throughout design, development, evaluation, and governance, rather than limiting input to post hoc review or ethical oversight.
In practice, this includes contributing to prompt design, interaction design, fine-tuning strategies, and evaluation protocol development, ensuring that behavioural science expertise and behavioural evaluation shape system behaviour from the outset rather than auditing it retrospectively.
3. **Fund behavioural evaluation explicitly (funders).**
Allocate dedicated resources for behavioural testing, simulation studies, and post-deployment monitoring.
This should include funding for scenario-based testing, experimental evaluation designs, and ongoing assessment of real-world user outcomes. Without explicit funding, behavioural evaluation is consistently deprioritised relative to technical performance metrics.
4. **Develop shared behavioural benchmarks and metrics (research community).**
Begin developing shared, theory-informed benchmarks that enable behavioural effects to be measured, compared, and replicated across systems.
This may include standardised scenario libraries, validated behavioural outcome measures, and agreed protocols for assessing constructs such as trust, reliance, decision quality, and emotional impact.
5. **Support interdisciplinary norms and training (institutions, funders).**
Encourage routine collaboration between AI researchers, behavioural scientists, clinicians, and domain experts through joint training programmes, interdisciplinary teams, and shared evaluation standards.
Embedding these norms within institutions and funding structures is essential to ensure behavioural science expertise and behavioural evaluation are consistently integrated into AI development.

## Conclusion

AI systems that interact with people inevitably influence behaviour. These effects are not incidental; they emerge through how AI systems communicate, personalise responses, and are used over time.

Behavioural science offers practical, well-established tools to understand and mitigate these effects. Treating behavioural science as foundational infrastructure for responsible AI supports the development of AI systems that are safer, more trustworthy, and more effective in real-world settings.

Embedding behavioural science expertise and evaluation early is likely to be more effective, and less costly, than responding to harm after deployment.

We hope this Open Letter contributes to a broader and more systematic approach to behavioural safety in AI and encourages continued collaboration across disciplines to ensure that AI systems are designed and governed with human behaviour firmly in view.

## Disclaimer

The views expressed in this article are those of the author(s). Publication in Wellcome Open Research does not imply endorsement by Wellcome.

## Data availability

No data are associated with this article.
