# Supplementary material for: The missing discipline in AI: a call for behavioural science
Source: Wellcome Open Res. 2026 Jun 15;11:152. Originally published 2026 Feb 26. [Version 2] doi: 10.12688/wellcomeopenres.25922.2 (PMC13555052; doi:10.12688/wellcomeopenres.25922.2)
Supplement: Peer review history

# Reviewer report: Reviewer response for version 2

**DOI:** 10.21956/wellcomeopenres.29500.r160636

**Published:** 2026-07-28

**Authors:** Kingsley Ofosu-Ampong

**Affiliations:** 1 Alliance of Bioversity International and International Center for Tropical Agriculture, Accra, Accra, Ghana

The letter is timely, clearly written, and well organized and sound. The central framing — AI systems as behavioural interventions deployed at scale — is useful and defensible, and the authors do commendable definitional work (behavioural science, behavioural safety, the distinction from research on agent behaviour). However, I have these three issues that I would like the authors to address;

 1. I see that the evidence bases and recommendations are implicitly **high-income-country in scope**, and this is unacknowledged. The cited empirical literature is drawn from settings with strong research and regulatory infrastructure, and the recommendations presume conditions — behavioural science capacity within AI teams, funders able to earmark evaluation budgets, governance structures with enforcement capability — that do not hold in many deployment contexts, particularly in low- and middle-income countries. This matters because behavioural risks are plausibly amplified in precisely those contexts: lower prior exposure for calibrating trust in machine-generated advice, interaction in second languages, mobile-first access, and thinner professional infrastructures whose substitution or delay the letter itself identifies as a harm. The letter need not solve this, but a paragraph acknowledging context-dependence and capacity asymmetries — and directing at least one recommendation toward it — would materially improve its global relevance and guard against the criticism that "foundational infrastructure" is being specified for a minority of the world's AI users.

 2. Also, I would like the authors to address the underrepresentation claim with empirical evidence. The assertion that behavioural science expertise is "often absent," "underrepresented," or "involved only informally and late" recurs throughout and rests repeatedly on the same reference cluster (refs 4–8). For a claim this central, the authors should provide at least one concrete anchor — a survey of AI team composition, an audit of evaluation practices, a documented case — or soften the language to reflect its currently anecdotal basis.

 3. Finally, I find that the named behavioural science tools are asserted, not demonstrated. The letter lists COM-B, the Behaviour Change Wheel, the Behaviour Change Technique Taxonomy, micro-randomised trials, and ecological momentary assessment, but doesn't illustrate what any of them would reveal about an AI system that standard evaluation misses.

Does the article adequately reference differing views and opinions?

Yes

Are all factual statements correct, and are statements and arguments made adequately supported by citations?

Partly

Is the Open Letter written in accessible language?

Yes

Where applicable, are recommendations and next steps explained clearly for others to follow?

Yes

Is the rationale for the Open Letter provided in sufficient detail?

Yes

Reviewer Expertise:

Behaviour and information systems scientist

I confirm that I have read this submission and believe that I have an appropriate level of expertise to confirm that it is of an acceptable scientific standard.

# Reviewer report: Reviewer response for version 2

**DOI:** 10.21956/wellcomeopenres.29500.r158255

**Published:** 2026-07-08

**Authors:** Sidra Sidra

**Affiliations:** 1 CSIRO, Eveleigh, Australia

The revised manuscript is much clearer and stronger than the first version. The authors have responded seriously to the earlier feedback. They now explain more clearly what they mean by behavioural science, provide more examples and evidence, recognise the contribution of related fields such as human–computer interaction and AI ethics, and describe psychological competence as an early idea rather than an established concept. The recommendations are also more detailed and identify who should take responsibility at different stages of AI development.

 A short table linking system characteristics, behavioural mechanisms, possible user outcomes, indicative risk levels, and suitable evaluation methods would strengthen both the definition of behavioural safety and the recommendations. For example, the authors could include a table with entries for different system features, as illustrated below:

**System feature:** Strongly personalised or adaptive systems.

**Possible behavioural mechanism:** Reinforcement of existing preferences, beliefs, habits, or vulnerabilities.

**Potential user outcome:** Narrowed perspectives, reduced autonomy, dependence, or harmful behavioural patterns.

**Indicative risk level:** Moderate to high.

**Suitable evaluation approaches:** Personalisation audits, factorial experiments, micro-randomised trials, SMART designs, comparisons across user profiles, longitudinal repeated-use studies, behavioural logging, and monitoring of reliance, agency, motivation, and reinforcing effects over time.

**What could be measured: **Changes in reliance, autonomy, motivation, confidence, diversity of information exposure, persistence of behaviour, dependence on the system, and whether effects strengthen or weaken over time.

 Similarly, this approach could be applied to other system features, such as frequent or long-term use, human-like or emotionally responsive design, persuasive language or repeated feedback, use by vulnerable populations, use in sensitive domains such as healthcare or finance, highly confident communication despite uncertainty, and systems where potential harms are difficult to reverse.

 Overall, the revised manuscript is a meaningful improvement.

Does the article adequately reference differing views and opinions?

No

Are all factual statements correct, and are statements and arguments made adequately supported by citations?

Partly

Is the Open Letter written in accessible language?

Yes

Where applicable, are recommendations and next steps explained clearly for others to follow?

Partly

Is the rationale for the Open Letter provided in sufficient detail?

Partly

Reviewer Expertise:

Human-AI collaboration

I confirm that I have read this submission and believe that I have an appropriate level of expertise to confirm that it is of an acceptable scientific standard.

# Reviewer report: Reviewer response for version 1

**DOI:** 10.21956/wellcomeopenres.28532.r151194

**Published:** 2026-04-16

**Authors:** Sidra Sidra

**Affiliations:** 1 CSIRO, Eveleigh, Australia

This Open Letter argues that behavioural science is a critically underrepresented discipline in the development, deployment, and governance of artificial intelligence systems.

 The authors begin from the observation that AI systems increasingly interact directly with people in high-stakes domains such as healthcare, education, finance, and social support. They argue that these interactions inevitably shape how people think, feel, decide, and act over time. They contend that the current landscape of responsible AI practice focuses predominantly on technical performance, bias, privacy, and security, and that this leaves a significant gap: the systematic evaluation of behavioural impacts.

 The letter identifies several specific mechanisms through which behavioural risks can emerge even when AI systems are technically accurate and functioning as intended. These include: automation bias, inappropriate trust calibration, anthropomorphism, emotional dependency, and the reinforcement of pre-existing beliefs through personalisation. The authors also highlight that language, framing, tone, and feedback loops embedded in AI interactions can subtly but meaningfully influence motivation, emotional regulation, and decision making.

 The authors conclude with five concrete recommendations directed at developers, deployers, funders, and research institutions: treat behavioural impact as a core evaluation criterion; embed behavioural expertise throughout the AI lifecycle; explicitly fund behavioural evaluation; develop shared behavioural benchmarks; and foster interdisciplinary norms and training. The letter closes by calling for ongoing collaboration across disciplines to ensure that human behaviour is placed at the centre of AI design and governance.

 The rationale is clearly stated but relies on assertion rather than evidence in several key areas, limiting its persuasive force for a sceptical reader. The letter repeatedly asserts that behavioural harms occur in practice but does not provide a single case study or documented incident. Even one worked example, for instance, an AI mental health chatbot that discouraged professional help-seeking, or a recommendation engine that narrowed a user's information environment, would substantially anchor the argument in reality. The authors should add at least two or three real-world examples, drawn from published literature or documented incidents, that demonstrate the kind of harm they are warning against.

 Additionally, A citation to empirical work, such as an audit of published AI evaluations or a survey of development teams, would significantly strengthen the claim that behavioural expertise is "often absent".

 The letter could engage with counterarguments and  alternative disciplinary perspectives. For example, Human-Computer Interaction (HCI), user experience (UX) research, and ethics review boards already address some of the concerns raised. The letter does not explain how behavioural science is distinct from, or additive to, these established practices.

 The scientific claims are broadly plausible and consistent with the literature, but citation density is insufficient relative to the scope and specificity of claims being made. Several critical assertions are unsupported. For example, the claim that "AI systems using "social heuristics and cognitive biases" including anthropomorphism and social mimicry to amplify influence", does not cite any references.

 The five recommendations are clearly structured and appropriately targeted. However, they remain at a level of aspiration rather than operational specificity, which limits their utility as a guide to action. Recommending existing models, training programmes, institutional structures, or funding schemes, that represent good practice and if they could be replicated or scaled, would be helpful.

Does the article adequately reference differing views and opinions?

No

Are all factual statements correct, and are statements and arguments made adequately supported by citations?

Partly

Is the Open Letter written in accessible language?

Yes

Where applicable, are recommendations and next steps explained clearly for others to follow?

Partly

Is the rationale for the Open Letter provided in sufficient detail?

Partly

Reviewer Expertise:

Human-AI collaboration

I confirm that I have read this submission and believe that I have an appropriate level of expertise to confirm that it is of an acceptable scientific standard, however I have significant reservations, as outlined above.

# Response

**Published:** 2026-05-15

**Authors:** Paul Sacher

**Affiliations:** Imperial College London Faculty of Medicine, London, England, UK

**Response to Reviewer 2** Manuscript Title:

 The missing discipline in AI: a call for behavioural science We thank the reviewer for their thoughtful and detailed review. We greatly appreciate the constructive feedback and believe it significantly strengthened the manuscript. All revisions are visible in the tracked-changes manuscript.

**Comment 1:**

**“The rationale is clearly stated but relies on assertion rather than evidence in several key areas…”** **Response:**

 We thank the reviewer for this important observation. In response, we substantially strengthened the empirical grounding of the manuscript throughout, particularly in the Introduction and “Where behavioural risks arise in practice” sections. The revised manuscript now incorporates additional empirical literature and examples related to:

 • automation bias,

 • socioaffective alignment,

 • conversational AI in mental health contexts,

 • recommendation systems,

 • psychological targeting,

 • anthropomorphic interaction with AI systems,

 • and behavioural reinforcement effects through personalisation and repeated interaction. We also revised several statements to ensure claims are appropriately calibrated to the current evidence base.

**Comment 2:**

**“The authors should add at least two or three real-world examples…”** **Response:**

 In response, we expanded the behavioural risks section to include more concrete empirical examples and application domains. These now include:

 • over-reliance on automated advice,

 • concerns related to conversational AI systems in mental health settings,

 • narrowing of information environments through recommendation systems,

 • and behavioural reinforcement through personalisation and framing. While we chose not to include extended case-study narratives in order to preserve the concise format of the Open Letter, we aimed to substantially improve the manuscript’s empirical grounding and practical concreteness.

**Comment 3:**

**“A citation to empirical work... would strengthen the claim that behavioural expertise is often absent.”** **Response:**

 We appreciate this suggestion and strengthened citation support throughout the manuscript, including in the Introduction and “What is currently missing” sections. We added literature related to:

 • responsible AI evaluation gaps,

 • AI impact assessment,

 • socioaffective alignment,

 • and behavioural science integration challenges in AI systems. We also revised wording in several locations to avoid overstating claims where evidence remains limited or emergent. **Comment 4:**

**“The letter could engage with counterarguments and alternative disciplinary perspectives…”** **Response:**

**We substantially revised the manuscript in response to this feedback.** The revised manuscript now explicitly discusses adjacent disciplines in the Introduction, including:

 • human–computer interaction (HCI),

 • user experience (UX) research,

 • and AI ethics. We clarify that behavioural science is intended to complement rather than replace these approaches. The revised text further explains how behavioural science contributes predictive and explanatory frameworks for understanding repeated interaction effects and longitudinal behavioural outcomes. **Comment 5:**

**“Several critical assertions are unsupported.”** **Response:**

 We carefully reviewed citation support throughout the manuscript and added multiple additional references and clarifications. This included strengthening support for claims related to:

 • anthropomorphism,

 • social heuristics,

 • persuasion and framing effects,

 • automation bias,

 • recommendation systems,

 • socioaffective alignment,

 • and conversational AI systems. We also revised several claims to ensure wording more accurately reflects the strength and limits of the current evidence base. **Comment 6:**

**“The recommendations remain at a level of aspiration rather than operational specificity…”** **Response:**

 We substantially expanded the operational specificity of the “What good practice looks like” and “Recommendations and next steps” sections. The revised manuscript now includes discussion of:

 • behavioural testing pipelines,

 • structured user scenarios,

 • simulation approaches using synthetic or simulated users,

 • exploratory stress-testing methods,

 • longitudinal evaluation,

 • behavioural benchmarks,

 • validated behavioural outcome measures,

 • post-deployment monitoring,

 • and interdisciplinary governance structures. We also expanded examples of where behavioural science expertise can contribute directly in practice, including prompt design, interaction design, evaluation protocol development, and monitoring of repeated interaction systems. These revisions were intended to address the reviewer’s concerns regarding operational clarity and practical implementation. We thank the reviewer again for their detailed and constructive feedback, which significantly improved the manuscript.

# Reviewer report: Reviewer response for version 1

**DOI:** 10.21956/wellcomeopenres.28532.r151752

**Published:** 2026-04-07

**Authors:** Daniel Bafour‑Koduah

**Affiliations:** 1 University of Cape Coast, Cape Coast, Ghana

1. the manuscript is built on behavioral harms and risks associated with AI systems however it lacks the empirical foundations to lay such claims. it is important the authors incorporate empirical evidence on the said issues demonstrating behavioral risks in practice. 

 2. there is lack of clarity on some operational concepts used: behavioral safety and psychological competence. the authors should provide clear definitions and their conceptualization of the concepts backed by literature.

 3. the arguments in the manuscript appears to be one-sided given that it lacks empirical evidence. kindly consider counterarguments and other critical perspectives to come out with strong argument.

 4. your recommendations are clear at the conceptual level. that is to say, it clearly outlines what can be done but practically, it fails to sufficiently outline how these recommendations can be put into practice. consider outlining a practical recommendation for AI practice.

 the manuscript is timely as it addresses a critical concern in today's world. the above concerns raised should be critically considered to produce a strong manuscript for indexing.

Does the article adequately reference differing views and opinions?

No

Are all factual statements correct, and are statements and arguments made adequately supported by citations?

Partly

Is the Open Letter written in accessible language?

Yes

Where applicable, are recommendations and next steps explained clearly for others to follow?

Partly

Is the rationale for the Open Letter provided in sufficient detail?

Partly

Reviewer Expertise:

Artificial Intelligence, Educational Technology, Curriculum and Instruction, Language Education, Socio-linguistics

I confirm that I have read this submission and believe that I have an appropriate level of expertise to confirm that it is of an acceptable scientific standard, however I have significant reservations, as outlined above.

# Response

**Published:** 2026-05-15

**Authors:** Paul Sacher

**Affiliations:** Imperial College London Faculty of Medicine, London, England, UK

**Response to Reviewer 1** Manuscript Title: The missing discipline in AI: a call for behavioural science We thank the reviewer for their thoughtful and constructive review. We greatly appreciate the feedback and have substantially revised the manuscript in response. All revisions are visible in the tracked-changes manuscript. **Comment 1:** “The manuscript is built on behavioral harms and risks associated with AI systems however it lacks the empirical foundations to lay such claims. It is important the authors incorporate empirical evidence on the said issues demonstrating behavioral risks in practice.”

 Response: We thank the reviewer for this important observation. In response, we substantially strengthened the empirical grounding of the manuscript throughout, particularly in the Introduction and “Where behavioural risks arise in practice” sections. The revised manuscript now incorporates additional empirical literature and examples related to: • automation bias and over-reliance on AI systems, • conversational AI and mental health contexts, • socioaffective alignment in human–AI interaction, • recommendation systems and narrowing of information environments, • psychological targeting, • anthropomorphic interaction with AI systems, • and behavioural reinforcement through personalisation and repeated interaction. We also revised several statements to ensure that claims are appropriately aligned with the strength of the current evidence base.

**Comment 2:** “There is lack of clarity on some operational concepts used: behavioral safety and psychological competence. The authors should provide clear definitions and their conceptualization of the concepts backed by literature.”

 Response: We appreciate this feedback and substantially revised both concepts for greater clarity and precision. These revisions were made primarily in the “Where behavioural risks arise in practice” and “What good practice looks like” sections. We now define behavioural safety as: “the extent to which an AI system identifies, reduces, and monitors predictable risks to users’decision-making, motivation, emotional wellbeing, sense of agency, and help-seeking behaviour over time.” We also revised and clarified the discussion of “psychological competence.” In the revised manuscript, the term is explicitly presented as a provisional organising concept rather than a validated construct. We further clarified that the concept refers to how AI-generated interactions shape reasoning, emotional regulation, behavioural decision-making, and interaction-level dynamics such as framing, tone, feedback, and perceived authority.

**Comment 3:** “The arguments in the manuscript appears to be one-sided given that it lacks empirical evidence. Kindly consider counterarguments and other critical perspectives to come out with strong argument.”

 Response: We thank the reviewer for this valuable suggestion. In response, we added explicit discussion of adjacent and complementary disciplines in the Introduction, including: • human–computer interaction (HCI), • user experience (UX) research, • and AI ethics. The revised manuscript now clarifies that behavioural science is intended to complement rather than replace these existing approaches. We further explain how behavioural science contributes additional predictive and explanatory frameworks for understanding repeated interaction effects over time.

**Comment 4:** “Your recommendations are clear at the conceptual level... however practically, it fails to sufficiently outline how these recommendations can be put into practice.”

 Response: We appreciate this important point and substantially expanded the practical specificity of both the “What good practice looks like” and “Recommendations and next steps” sections. The revised manuscript now includes discussion of: • behavioural testing pipelines, • structured user scenarios, • simulation approaches using synthetic or simulated users, • exploratory stress-testing methods, • longitudinal evaluation, • behavioural benchmarks, • behavioural outcome measures, • post-deployment monitoring, • and interdisciplinary governance structures. We also expanded discussion of where behavioural science expertise can contribute directly in practice, including prompt design, interaction design, evaluation protocol development, and monitoring of repeated interaction systems. We thank the reviewer again for their *th*oughtful feedback, which substantially strengthened the manuscript.
